# Topical and Targeted Delivery of siRNAs to Melanoma Cells Using a Fusion Peptide Carrier

**DOI:** 10.1038/srep29159

**Published:** 2016-07-04

**Authors:** Renquan Ruan, Ming Chen, Sijie Sun, Pengfei Wei, Lili Zou, Jing Liu, Dayong Gao, Longping Wen, Weiping Ding

**Affiliations:** 1 Center for Biomedical Engineering, University of Science and Technology of China, Hefei, Anhui 230027, China; 2 Department of Electronic Science and Technology, Hefei, Anhui 230027, China; 3 School of Life Sciences, University of Science and Technology of China, Hefei, Anhui 230027, China; 4 Fujian Longsheng Biotechnology (Group) Co., Ltd., Longyan, Fujian 364000, China; 5 Department of Pharmacology, Anhui University of Chinese Medicine, Hefei, Anhui 230038, China; 6 Department of Laboratory Medicine, University of Washington, Seattle, WA 98195, USA; 7 Department of Mechanical Engineering, University of Washington, Seattle, WA 98195, USA

## Abstract

Topical application of siRNAs through the skin is a potentially effective strategy for the treatment of melanoma tumors. In this study, we designed a new and safe fusion peptide carrier SPACE-EGF to improve the skin and cell penetration function of the siRNAs and their targeting ability to B16 cells, such that the apoptosis of B16 cells can be induced. The results show that the carrier is stable and less toxic. The EGF motif does not affect the skin and cell penetration function of the SPACE. Because EGF can strongly bind EGFR, which is overexpressed in cancer cells, the targeting ability of the SPACE-EGF-siRNA complex is increased. *In vitro* experiments indicate that GAPDH siRNAs conjugated with SPACE-EGF can significantly reduce the GAPDH concentration in B16 cells, and c-Myc siRNAs can cause the gene silencing of c-Myc and thus the apoptosis of cells. *In vivo* experiments show that the topical application of c-Myc siRNAs delivered by SPACE-EGF through the skin can significantly inhibit the growth of melanoma tumors. This work may provide insight into the development of new transdermal drug carriers to treat a variety of skin disorders.

Melanoma is one of the most serious types of malignant tumors in the world, and it causes the majority of deaths (~75%) related to skin cancer^1^. Melanoma begins in the melanocytes, which may occur anywhere in the skin. The routine treatment is to remove the tumor by surgery. However, the likelihood of relapse depends on how deeply the tumor has invaded the skin layer. Some improved therapeutic options such as chemotherapy and immunotherapy have recently emerged, but the therapeutic outcome is still limited because melanoma has been proven to be resistant to most available chemotherapy and immunotherapy^2^,^3^. siRNA therapy (siRNA: small interfering RNA) is a promising strategy for cancer treatment, with reduced toxicity compared to that of conventional therapies^4^. Combination therapy using siRNA and one or more chemotherapy drugs may be conductive to decreasing the required dose of the drug and improving the therapeutic effect. Today, various siRNA-based chemotherapies have been developed to treat melanoma mostly via intravenous, intramuscular and oral administrations^5^,^6^,^7^. For example, siRNAs against N-Ras, c-Myc, and vascular endothelial growth factor (VEGF) can inhibit tumor growth in B16F10 melanoma lung metastasis^7^,^8^.

Currently, the topical application of siRNAs to treat tumors is of particular interest^9^. However, this approach faces two challenges. The first challenge is how to deliver the macromolecular siRNAs into skin. Skin is a highly effective defensive barrier designed to protect organisms. It is believed that skin prevents macromolecules (>500 Da) from entering bodies. siRNAs are negatively charged, hydrophilic, and relatively large (~13 kDa). These characteristics cause the poor penetration of siRNAs into skin and thus the low absorption by melanoma cells. Traditionally, siRNA delivery is assisted by lipophilic analogs, such as liposomes^10^. However, the lipophilic analogs are sometimes toxic to cells^11^. As for the development of safe delivery systems, such as gold nanoparticles^12^ and polymer materials^13^, they still confront the size-induced drug delivery difficulty across the skin. In recent years, biologically inspired peptides, such as SPACE (skin penetrating and cell entering peptide) and TD1 (ACSSSPSKHCG), discovered using the phage display technique, have been used to facilitate the delivery of macromolecular drugs into the skin^14^,^15^,^16^,^17^. SPACE carries cargos across viable cells, whereas TD1 temporarily opens the skin barrier in an energy-dependent fashion to mediate the transport of macromolecules across skin^18^. These short peptides make it possible to deliver siRNAs via skin to melanoma cells; however, related work has seldom been reported. Only Lin *et al.* used TD1 to enhance the delivery of siRNAs across the rat footpad skin to knock down a targeted gene^19^.

The second challenge is how to target melanoma cells. Epidermal Growth Factor Receptor (EGFR) is an attractive target in many cancer cells. The number of EGFR molecules may reach one hundred thousand in one glioma cell, whereas there are only a few thousand in one healthy cell such as a keratinocyte, fibrocyte, or endotheliocyte^20^. In primary melanoma and cutaneous metastasis, EGFR levels were approximately 13-fold higher compared with those of normal melanocytes^21^. EGFR binds EGF with high affinity, and the EGF-EGFR complex can be internalized efficiently by receptor-mediated endocytosis^22^; as a result, EGFR is an excellent target for fusion proteins with macromolecular drugs. For example, the doxorubicin-peptide conjugate has been used for targeted delivery to EGFR over-expressing tumor cells^23^ and the diphtheria toxin-EGF fusion protein has been used to effectively kill glioma cells^24^.

In sum, topical application of siRNAs through the skin is a potentially effective strategy for the treatment of melanoma. However, due to the poor permeation of siRNAs across the stratum corneum, the weak targeting ability of siRNAs to cancer cells and thus the low penetration of siRNAs into melanoma cells, this strategy has not been successfully practiced. In this work, our objective was to develop a peptide-based carrier protein to improve the permeation of siRNAs across the stratum corneum, to promote the targeted delivery of siRNAs to cancer cells, and to increase the penetration of siRNAs into cancer cells. The carrier we constructed is SPACE-EGF. Because SPACE can penetrate skin and cells and EGF can target the EGFR overexpressed in cancer cells, SPACE-EGF is expected to carry siRNAs into B16 cells, which would then induce the apoptosis of B16 cells. We experimentally confirmed the function of SPACE-EGF and the delivery of GAPDH and c-Myc siRNAs mediated by SPACE-EGF across the skin and into B16 cells. This work is of significance for the development of transdermal macromolecular drug carriers.

## Results and Discussion

### Construction of SPACE-EGF and SE-siRC

**Figure 1:**
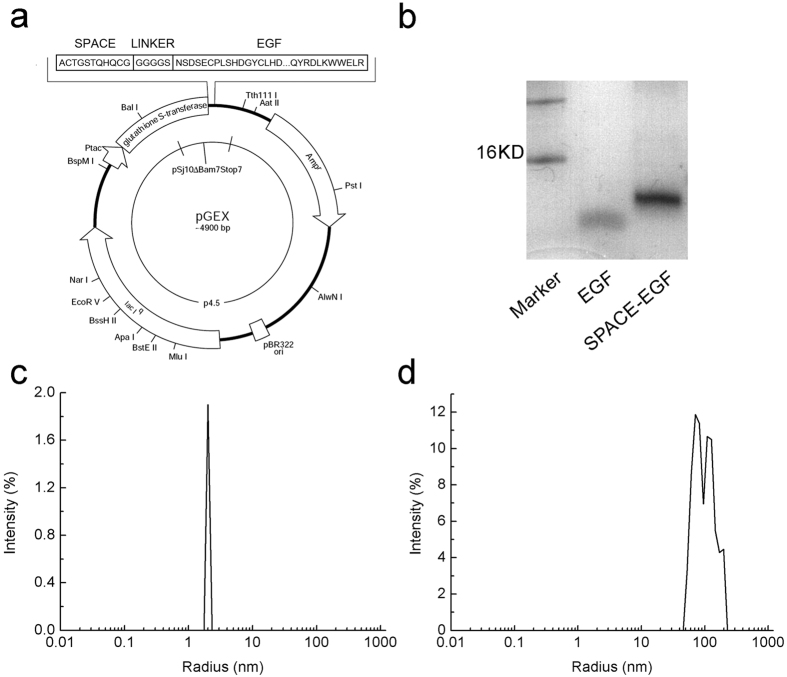
Determination of the characteristics of SPACE-EGF. (**a**) The clone construction of SPACE-EGF in pGEX-6P-1. (**b**) The protein purification detected by SDS-PAGE. (**c**) The size distribution of SPACE-EGF. (**d**) The size distribution of the SPACE-EGF-siRNA complex (SE-siRC). The linked siRNA was GAPDH-siRNA.

SPACE-EGF is a fusion protein of EGF and SPACE interconnected via a glycine-serine linker (GGGGS). In the experiment, a DNA fragment coding for SPACE-EGF was obtained by PCR amplification, and the PCR fragment was then inserted into pGEX-6p-1 vector (Fig. 1a). SPACE-EGF was expressed in *E. coli* and purified by a GST purification system to more than 95% homogeneity as judged by SDS electrophoresis (Fig. 1b). The protein sequence was identified by the LD-LC-MS. SPACE-EGF is a dimer in solution, as is the activated EGF. Its radius is approximately 2 nm (Fig. 1c). Because siRNA is a linear macromolecule, the size of the SPACE-EGF-siRNA complex (SE-siRC; the radius is approximately 50–200 nm) is much larger than SPACE-EGF and the size distribution of SE-siRC is also wide (Fig. 1d). In practice, to reduce the size of SE-siRC, increase the size uniformity, and then facilitate the delivery of SE-siRC across skin and into cancer cells, one may improve the conjugation process or purify the complex.

### Stability and Cytotoxicity of SPACE-EGF

**Figure 2:**
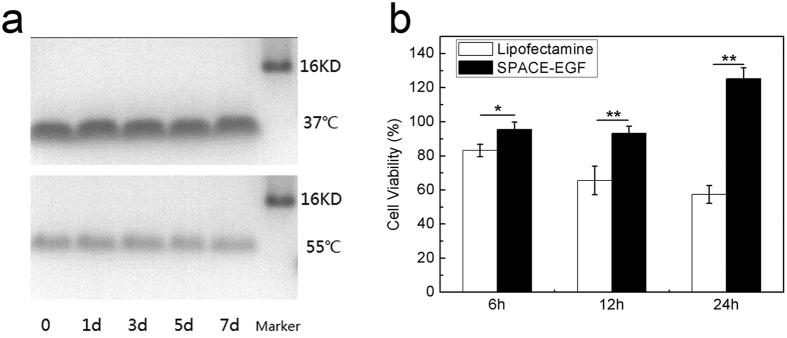
Stability and cytotoxicity of SPACE-EGF. (**a**) SPACE-EGF was incubated for 1, 3, 5 and 7 days at 37 °C and 55 °C. (**b**) The viabilities of MEF cells treated with 1 mg/mL of SPACE-EGF or 1 μM of Lipofectamine 2000 for 6, 12 and 24 hours. The experiments were repeated at least 3 times.

The stability of the vector SPACE-EGF was investigated at 37 °C and 55 °C in 7 days in terms of protein degradation. The cytotoxicity was compared with the traditional transfection agent Lipofectamine 2000. The SDS-PAGE shows a very slight change when the temperature varies from 37 °C to 55 °C (Fig. 2a). An MTT assay shows that SPACE-EGF is less toxic to mouse embryo fibroblast (MEF) cells even at a high concentration of 1 mg/mL (Fig. 2b). It should be noted that the ligand of EGF could promote cell proliferation by binding EGFR; as a result, the viability of cells when SPACE-EGF is applied may be over 100% (Fig. 2b). However, the drug siRNA carried by SPACE-EGF mainly determines the cell viability. In this work, the vector designed is very small and thus can pass through kidney, but for the route of administration via the skin, the drug excretion through kidney is often little. Here, we have confirmed that the vector is less toxic to MEF cells. The safety of the vector for other cells still needs to be confirmed in the future study.

### Skin Penetration of SPACE-EGF and SE-siRC

**Figure 3:**
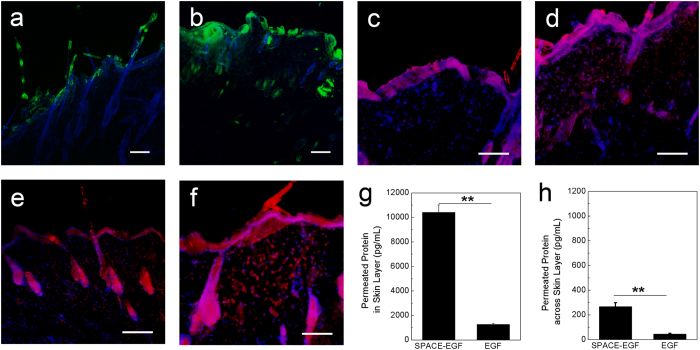
Skin penetration of SPACE-EGF and SE-siRC. (**a**) The skin penetration of EGF (20 μg) after 4 hours. (**b**) The skin penetration of SPACE-EGF (20 μg) after 4 hours. (**c**) The skin penetration of EGF-siRNA after 8 hours. (**d**) The skin penetration of siRNA after 8 hours with co-administered Lipofectamine 2000. (**e**) The skin penetration of SPACE-siRNA after 8 hours. (**f**) The skin penetration of SE-siRC after 8 hours. (**g**) The permeated SPACE-EGF or EGF in the skin layer after 16 hours. (**h**) The permeated SPACE-EGF or EGF across the skin layer after 16 hours. The linked siRNA was GAPDH-siRNA. ELISA assay was used to quantify the permeated protein. Scale bar: 40 μm. The experiments were repeated at least 3 times.

To use SPACE-EGF for carrying siRNA into cancer cells via the skin, we should first confirm the hypothesis that the fusion protein mode does not affect the skin penetration function of SPACE. We examined the *in vitro* penetration ability of SPACE-EGF through the abdomen skin of SD rats. Here, FITC-labeled EGF (Green Fluorescence in Fig. 3a,b) was used to observe the EGF or SPACE-EGF delivery. Cy-3-labeled GAPDH siRNA (Red Fluorescence in Fig. 3c–f) was used to observe the siRNA delivery. Our results show that SPACE-EGF can still significantly penetrate into the skin (Fig. 3a vs Fig. 3b) and it enhances the penetration of siRNA (Fig. 3c–f) via the SPACE-EGF-siRNA complex.

In this work, as the cell apoptosis induced by the delivered siRNA is expected, we should further confirm whether the permeated protein accumulates in the skin layer. Our results show that the fusion peptide carrier SPACE-EGF makes most proteins (EGF) stay in the skin layer (Fig. 3g). In addition, our results also indicate that when EGF is applied, the permeated protein (EGF), whether in or across the skin layer, is far less than when SPACE-EGF is used (Fig. 3g,h). The results here suggest that the EGF motif does not affect the function of SPACE in terms of enhancing drug delivery across the skin.

### Cell Penetration and Cell Targeting of SPACE-EGF or SE-siRC

**Figure 4:**
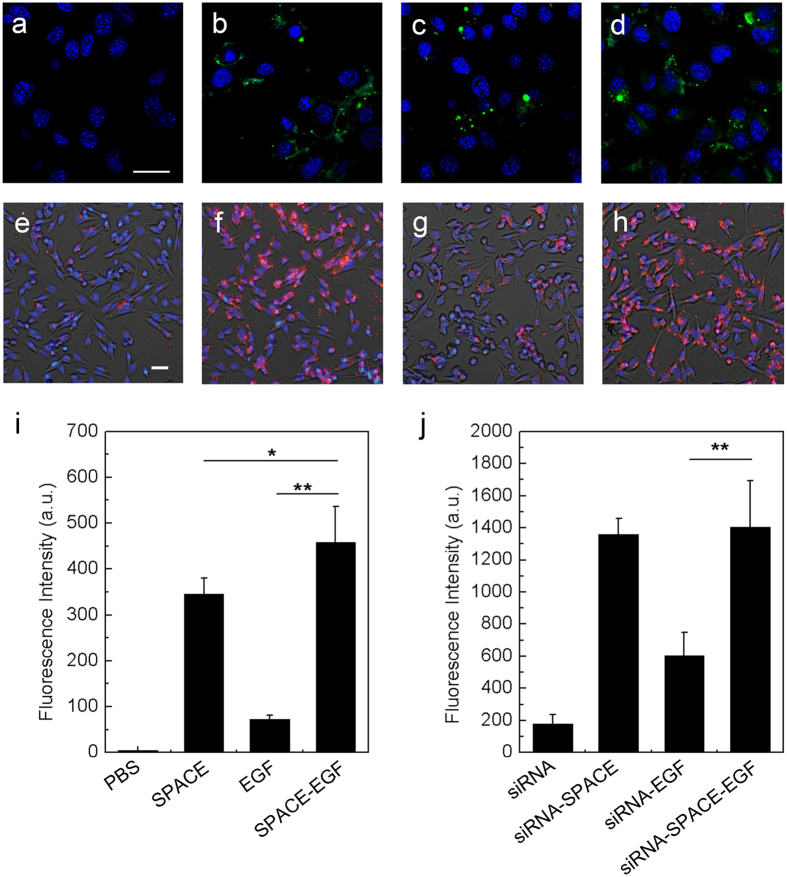
Cell penetration of SPACE-EGF and SE-siRC. In (**a–d**) cells were treated for 6 hours with PBS, SPACE, EGF, and SPACE-EGF, respectively. In (**e–h**) cells were incubated for 6 hours with siRNA, siRNA-SPACE, siRNA-EGF, and siRNA-SPACE-EGF, respectively. In (**b**) SPACE was also labeled fluorescently. In (**i,j**) the mean fluorescence intensities in melanoma cells after 6 hours were compared. Scale bar: 20 μm. The linked siRNA was GAPDH-siRNA. The experiments were repeated at least 5 times.

In the previous section, we found SPACE-EGF/SE-siRC accumulated in the skin layer. Next, we further confirmed the cell penetration ability of SPACE-EGF/SE-siRC. In the experiments, we investigated the internalization of SPACE, EGF, and SPACE-EGF in viable cells. The results again show that the EGF motif does not alter the cell penetration function of SPACE (Fig. 4a–d). The extent of internalization of SPACE-EGF in B16 cells is highest among all groups (Fig. 4i). In term of the delivery of SE-siRC into cells, the conclusion we drew is consistent with the above (Fig. 4e–h,j): SPACE-EGF can carry cargo proteins into cells.

**Figure 5:**
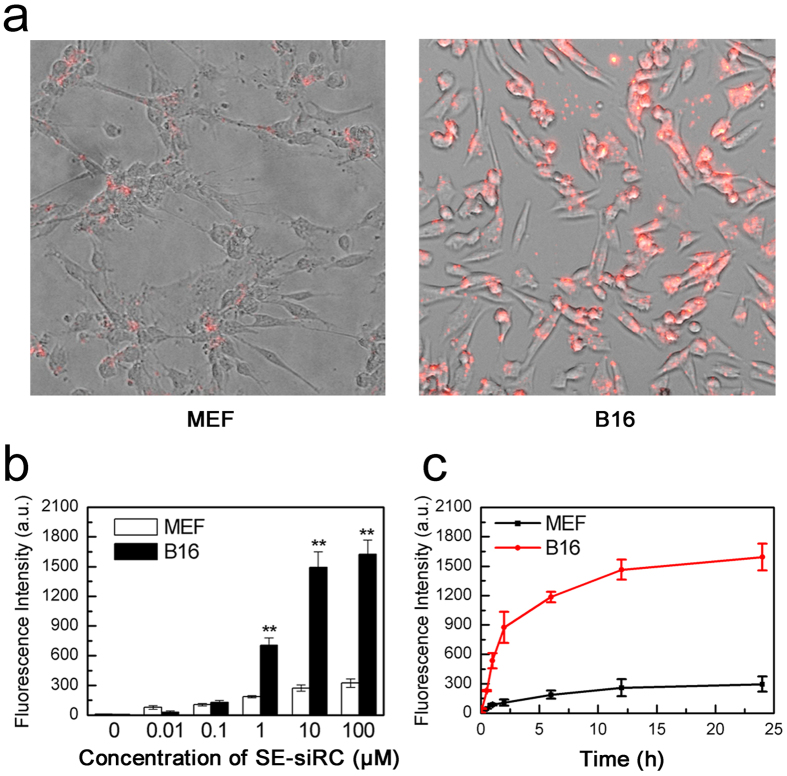
Targeting of SE-siRC to melanoma cells. (**a**) Representative images of the targeting of SE-siRC to B16 cells with overexpressing EGFR and mouse embryo fibroblast cells (MEF) after treating for 1 hour with 10 μM of SE-siRC. (**b**) Fluorescence intensities of cells treated with various concentrations of SE-siRC for 12 hours. (**c**) Binding efficiencies at various times with 10 μM of SE-siRC. The linked siRNA was GAPDH-siRNA. The experiments were repeated at least 5 times.

In this work, due to the EGF motif, the targeting ability of SPACE-EGF to cancer cells is expected. The results show that SPACE-EGF can rapidly localize to EGFR-overexpressing melanoma cells after 1 h of treatment; however, mouse embryo fibroblast cells (MEF) in the control group have a low binding efficiency to SE-siRC (Fig. 5a). In addition, the binding efficiency shows a dose and time dependence (Fig. 5b,c). The results show that when the concentration of SE-siRC is larger than 10 μM or the treatment time is extended to more than 12 hours, the binding efficiency increases very slowly. Together, SPACE may facilitate the internalization of carried proteins via micropinocytosis at a certain concentration^14^, and EGF can bind EGFR with a high affinity and penetrate into cells by EGFR internalization endocytosis^22^. Therefore, SPACE-EGF can increase the targeting ability of siRNA to B16 cells, the penetration of siRNA into B16 cells, and the internalization of siRNA in B16 cells.

### Gene Silencing in B16 cells with SE-siRC

**Figure 6:**
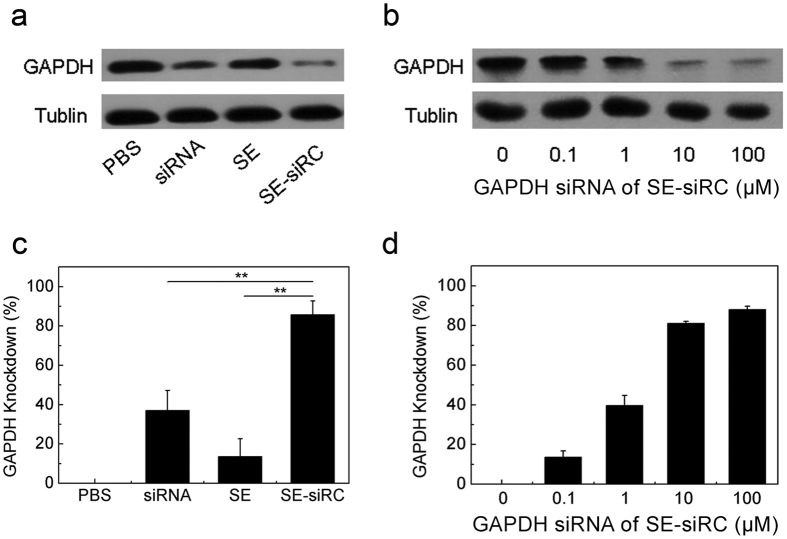
GAPDH silencing by SE-siRC. (**a,c**) The knockdown degree of the GAPDH protein in B16 cells treated for 48 hours with 10 μM GAPDH SE-siRC, PBS, siRNA, or SE. (**b,d**) The expression of GAPDH under various concentrations of GAPDH siRNA. The experiments were repeated at least 3 times.

To confirm gene silencing in cancer cells with siRNAs transported by SPACE-EGF, first we studied the GAPDH expression in B16 cells upon the addition of GAPDH siRNAs. GAPDH is a housekeeping protein as well as a common siRNA target. In this study, the knockdown of GAPDH in B16 cells was determined by a Western Blot analysis. SPACE-EGF works as a vector to transport siRNA into cells via micropinocytosis. Then, siRNA is released from the complex after lysosomal degradation in cells (it can be detected at 260 nm in a UV visible spectroscopy). The results show that GAPDH SE-siRC causes a significant reduction in the GAPDH level compared to the control groups such as PBS, siRNA, and SE (Fig. 6a,c). Furthermore, the knockdown degree increases with the concentration of GAPDH siRNA (Fig. 6b,d). Ten μM GAPDH siRNA causes approximately 80% of the GAPDH knockdown.

**Figure 7:**
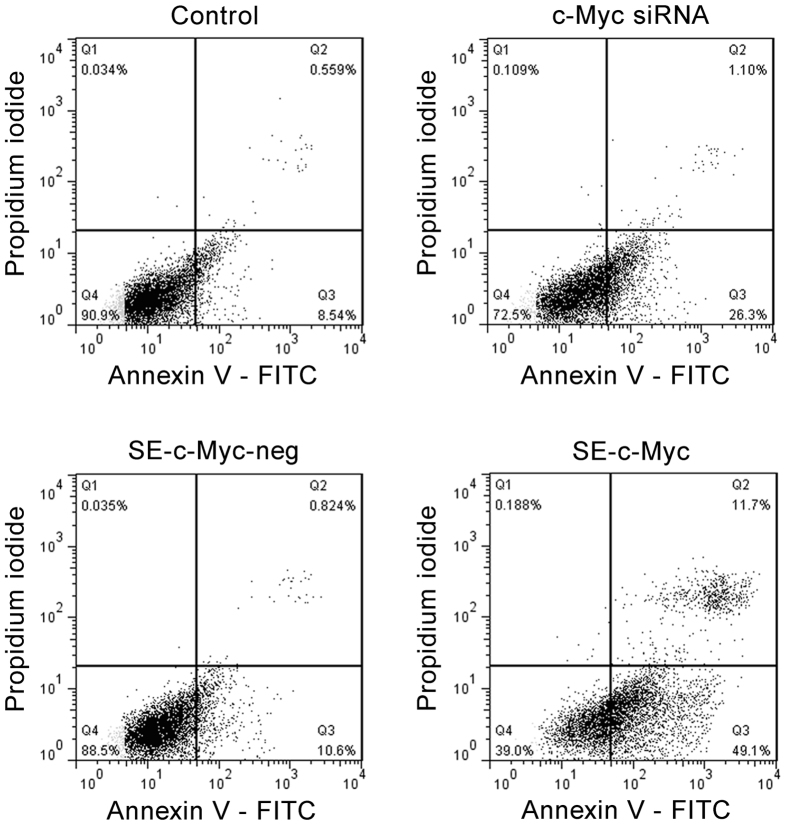
Induction of apoptosis of melanoma cells by SPACE-EGF-mediated c-Myc siRNAs. B16 cells were incubated with 10 μM of c-Myc siRNAs, negative c-Myc SE-siRC and c-Myc SE-siRC for 48 hours, and then the apoptosis was assessed by the flow cytometry analysis. In this work, early apoptotic (Annexin V+/PI−), late apoptotic/necrotic (Annexin V+/PI+), and live (Annexin V−/PI−) populations were clearly identified.

Then, we investigated the gene silencing of c-Myc mediated by SPACE-EGF. The c-Myc is related to the proliferation of melanoma cells and has been found to be overexpressed in melanoma cells^25^,^26^,^27^. The c-Myc depletion could inhibit cell proliferation, causing cell cycle arrest and inducing the cellular apoptotic response associated with activation of caspase-3^27^. In this work, we determined the effect of c-Myc siRNAs mediated by SPACE-EGF on the apoptosis of cells treated with 10 μM of SE-siRC for 48 hours and stained by Annexin V and PI. The results show that the apoptosis rate of cells in the c-Myc SE-siRC group is significantly higher than the one in the c-Myc siRNA group (Fig. 7). SPACE-EGF mediated c-Myc siRNA results in 49.1% Annexin V positive and PI negative cells whereas c-Myc siRNA only results in 26.3%. The results here indicate that SPACE-EGF increases the permeation of c-Myc into cells and causes the apoptosis of cells.

### Growth Inhibition of Tumors with SE-siRC

**Figure 8:**
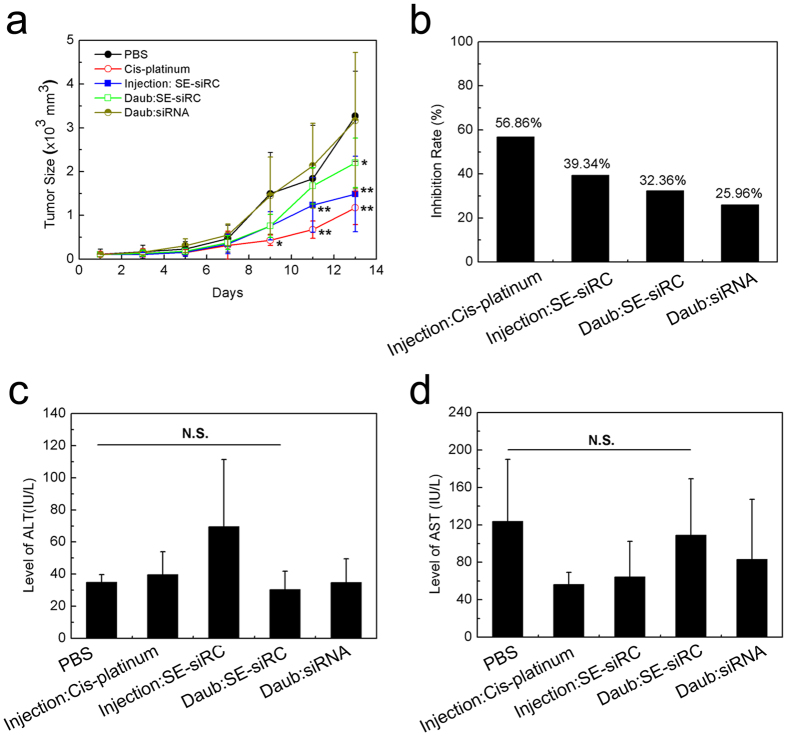
Growth inhibition of B16F10 tumors and hepatotoxicity of SE-siRC (n ≥ 6). (**a**) The size of tumors under various formulations. (**b**) The growth inhibition rate of B16F10 tumors with SE-siRC. (**c**) The level of ALT in the mice after the treatment with SE-siRC. (**d**) The level of AST in the mice after the treatment with SE-siRC. The linked siRNA was c-Myc siRNA. N.S.: No Significance.

To confirm the antineoplastic activity of c-Myc siRNA mediated by SPACE-EGF, we performed *in vivo* experiments to investigate the tumor growth-inhibitory effect of SE-siRC in the tumor-bearing mice (Fig. 8). The results show that when only the c-Myc siRNA is daubed on the skin, the growth of tumors is not inhibited as the average tumor size in the c-Myc siRNA group is very close to the one in the PBS group (Fig. 8a). However, when SE-siRC is injected subcutaneously or daubed topically on the skin, the tumor growth-inhibitory effect after 13 days is significant (the tendency of the tumor growth suppression presented here is consistent with the one when the c-Myc siRNA is delivered by the targeted nanoparticles through intravenous injection)^28^. Compared to the cis-platinum group, although the inhibition rates for both injection and daub of SE-siRC are lower, they still reach 39.34% and 32.36%, respectively (Fig. 8b).

In this study, we adopted a high concentration of c-Myc siRNA to make results significant according to the literature^28^. In the delivery of SE-siRC through the skin, because of a high frequency of administration, the cumulative dose of c-Myc siRNA on the skin reaches 31.2 mg/kg mouse. However, the dose received actually by rats is far less than the value. The reason is that the efficiency of the delivery of macromolecular drugs across skin is usually low even in the presence of drug carriers^15^. Here, the size of some complexes is large and then they are hardly delivered through the skin. Consequently, the quantity of c-Myc siRNA delivered through the skin is lower than through the injection and thus, the inhibition rate in the SE-siRC daub group is lower than in the SE-siRC injection group (Fig. 8a).

In addition, under the dose and frequency of administration adopted here, compared to the PBS group, the levels of ALT (Fig. 8c) and AST (Fig. 8d) in the SE-siRC daub group are not changed significantly, which implies no hepatotoxicity for the administration of SE-siRC via the skin in the treatment of the subcutaneous melanoma tumors (the topical application of SE-siRC via the skin not only avoids the significant first-pass effect through the liver, but also reduces the toxicity of SE-siRC to the liver).

The antineoplastic activity of c-Myc siRNA delivered by SPACE-EGF through skin is promising. However, an intensive study still needs to be conducted in the future. First, one should develop an optimized dose and frequency of administration for the treatment of subcutaneous tumors. In this work, the inhibition rate of the tumor growth is low due to a small quantity of SE-siRC across the skin. Second, one should further investigate the safety of the therapeutic method. Here, no hepatotoxicity is shown under a small quantity of SE-siRC delivered through the skin. It does not, however, mean that there is always no toxicity to the liver, especially when the quantity of SE-siRC delivered is increased. In addition, one also needs to study the toxicity to other organs. Third, one should identify a motif more specific to cancer cells. The carrier SPACE-EGF designed here although has a significant targeting ability to EGFR in cancer cells, may bind healthy cells and then cause uncertain consequences. Therefore, the EGF motif is not ideal, and a motif specific to cancer cells is expected in practice.

## Conclusions

In this study, we, for the first time, designed a fusion peptide carrier SPACE-EGF to facilitate the delivery of siRNAs into cancer cells through skin and thus silence related genes to kill these cells. *In vitro* experiments have confirmed the SPACE motif-induced penetration function across the skin and into cancer cells and the EGF motif-induced targeting ability to cancer cells. With the help of SPACE-EGF, conjugated GAPDH and c-Myc siRNAs can be delivered into targeted cancer cells, knocking down related genes and inducing the apoptosis of cancer cells. *In vivo* experiments have confirmed that siRNA can be delivered topically through the skin and targeted to the subcutaneous melanoma tumors using the fusion peptide carrier SPACE-EGF, and then the growth of tumors can be inhibited.

The study reported here offers a strategy to treat melanoma by delivering siRNAs into melanoma cells through the skin. In the strategy, the fusion protein can be designed to target any cells; as a result, siRNA therapy can be extended to treat a variety of skin disorders, such as epidermal hyperplasia, cutaneous inflammation, and skin cancer.

## Methods

### Ethics Statement

All animal experiments were conducted in accordance with relevant guidelines and regulations of the Medical Ethics Committee at the University of Science and Technology of China (USTC). All rats were bred and housed at the Association of Laboratory Care (an accredited facility at the School of Life Sciences, USTC), where the conditions are specific pathogen-free. All protocols were approved by the Medical Ethics Committee at USTC (authorization number: USTCACUC1501017).

### Cell Culture

A mouse melanoma cell line (B16-F10) and mouse embryo fibroblasts (MEF) (Cell Bank, Shanghai, China) were cultured continuously at 37 °C and 5% CO2 in Dulbecco’s modified Eagle’s medium (DMEM; Gibco, USA) supplemented with 10% fetal bovine serum (FBS; Gibco, USA) and 1% penicillin-streptomycin (Gibco, USA)^18^. Cell culture dishes from MatTek Co. (Ashland, MA, USA) were rinsed three times with phosphate buffered saline (PBS) before use.

### Construction of Clone Vector

The DNA sequences of EGF (PTGlab, Wuhan, China), SPACE and the linker were connected and amplified by two-turn polymerase chain reaction (PCR). Primers were synthesized from Sangon Biotech Co., Ltd., Shanghai, China. In the first turn, the forward and reverse primers were 5′GCATCAGTGTGGTGGCGGCGGTGGTTCTAATAGTGACT3′ and 5′CCGCTCGAGCTAGCGCAGTTCCCACCACTTC3′, respectively. In the second turn, the reverse primer was still 5′CCGCTCGAGCTAGCGCAGTTCCCACCACTTC3′, but the forward primer was changed to 5′CATGCCATGGGCATGTACCGGTAGCACCCAGCATCAGTGTGG3′. The fusion gene fragments of SPACE-EGF were inserted into the pGEX-6p-1 vectors through two restriction sites (EcoR I and Xho I). A recombinant reaction was run at 16 °C overnight and the recombinant plasmid was transformed into Origami competent cells (Novagen, Germany) for expression.

### Purification of SPACE-EGF

The *E. coli* strain harboring the expression plasmid was cultured at 37 °C overnight in LB medium containing 100 μg/mL of ampicillin. The bacteria were diluted 1:100 in TB medium and then incubated at 16 °C for 20 h with rigorous shaking. After harvesting and re-suspending the cell pellet in PBS buffer, the soluble protein was extracted from the bacteria with high-pressure treatment. One hundred microliters of cell lysate were incubated with 10 mL of equilibrated GST resin (GE Healthcare, USA) at 4 °C overnight. The resin was washed with 20 times its volume of PBS buffer, followed by treatment with 100 IU of TEV protease for 12 h at 4 °C. The cleaved recombinant protein was collected after centrifugation at 6,000 × g for 10 min.

### Identification of SPACE-EGF and SE-siRC

The protein concentration was determined by a BCA assay (Beyotime, Beijing, China). The protein molecular weight and purity were estimated by SDS/PAGE and were verified by Liquid Desorption-Liquid Chromatography Mass Spectrometry (LD-LC-MS). The molecular size was detected by Dynamic Light Scattering (DLS; DynaPro-MS800, USA). Technical assistance for the DLS and LD-LC-MS was supplied by the Research Centre for Life Sciences at USTC.

### Peptide Synthesis

The peptide sequence for the SPACE peptide control was ACTGSTQHQCG with the formation of the disulfide bond between the cysteines to produce a cyclic peptide. Fluorescein isothiocyanate (FITC)-conjugated SPACE-EGF, EGF and SPACE were synthesized by Biox-Vision Inc., Hefei, China. The dye was placed at the N-terminus of the peptide and protein.

### Conjugation of siRNA and SPACE-EGF

A conjugation experiment was performed as described in the literature^17^. Two milligrams of SPACE-EGF was incubated with the 10 mM MES [2-(N-morpholino) ethanesulfonic acid] buffer containing 10 mM of EDAC [N-(3-Dimethyl aminopropyl)-N′-ethylcarbodiimide hydrochloride] and 10 mM of NHS [N-hydroxysulfosuccinimide sodium salt] (MES, EDAC and NHS were from Sangon Biotech Co., Ltd., Shanghai, China) for 15 min. The amine-modified GAPDH siRNA (5′-Cy–GACGUAAACGGCCACAAGUUC-3′) or the amine-modified mouse c-Myc siRNA (5′-GAACAUCAUCAUCCAGGAC-3′) from Genepharma (Shanghai, China) was then added to the mixture to conjugate the peptide to the siRNA and allowed to mix overnight.

### Cell Penetration Study

The cell penetration experiment was performed according to previous literature reports^14^,^17^. Cells (∼1.2 × 10^4^) were seeded on a glass culture dish with a bottom coated with poly-D-lysine. After incubation at 37 °C for 12 h, the media was removed, and then 20 μL fluorescent peptides were mixed with 180 of μL media and subsequently added to the cell culture dish. For the control group, 20 μL of PBS was replaced with a peptide solution. Cells were washed with PBS after incubation at 37 °C and then incubated with 1% trypan blue for 5 min to quench fluorescence on the surface of cells. After that, the cells were fixed with 4% paraformaldehyde for 3 min and washed again in PBS. Next, the cells were incubated with Hoechst 33342 (5 μg/mL) for 5 min and then washed in PBS. Finally, the cell culture dish was filled with PBS and was imaged using a confocal fluorescence microscopy (LSM510; Zeiss, Oberkochen, Germany). Images were processed using Image J.

### Skin Penetration Study

The skin penetration study was conducted as described in our previous work^16^. Briefly, the skin without subcutaneous fat was isolated from the abdomen of SD rats (200 ± 10 g) and visually inspected for any defects before the skin penetration study. In experiments, the isolated skin was sandwiched between the upper and lower parts of Franz diffusion cells (5 mL receptor slot; 0.5 cm^2^ diffusion area; PermeGear, USA). Five hundred microliters of the freshly prepared drug formulations containing the indicated protein was added to the donor compartment. Two hundred microliters of samples from the receptor compartment were taken at desired times (the same volume of saline was replaced after the sample was taken). The protein concentration in the receptor slot was determined using an EGF ELISA assay.

### Cytotoxicity of SPACE-EGF

The cytotoxicity of SPACE-EGF was determined using an MTT cell proliferation assay (Beyotime, Beijing, China). Cells were incubated with 1 mg/mL of SPACE-EGF in media for 6, 12, or 24 h. Untreated cells with liposome (Invitrogen, USA) were used as the control group. Viability was determined using a microplate reader (ELx800; Biotech Instruments Inc., USA) according to the manufacturer’s instructions.

### *In Vitro* Delivery of siRNA

The SPACE-EGF-siRNA complex was added to the appropriate cell culture media to obtain a final concentration of 10 μM of siRNA^14^. The siRNA concentration was determined by an ultraviolet spectrophotometer (Beckman Coulter DU640; CA, USA). SE-siRC was then added to the cells and allowed to incubate for 24 h. Cells were imaged using a confocal microscopy. The fluorescence spectrophotometer (F97XP; Lengguang Tech., Shanghai, China) was used to determine fluorescence intensity.

### *In Vitro* Gene Silencing

Cells were seeded in the 96-well plates (∼10,000 cells/well) (Corning Inc., NY, USA) and allowed to attach overnight. The cells were then incubated with 100 μL of 10 μM siRNA, 10 μM SE-siRC, 10 μM siRNA-SPACE, or 1 mg/mL SPACE-EGF. After 48 h, GAPDH was measured using the GAPDH Assay Kit (Life Technologies, Grand Island, NY, USA). A Western Blot assay was used to analyze the knockdown efficiency.

### Cell Apoptosis

Apoptotic cells were quantified using an Annexin V-FITC Apoptosis Kit I (Beyotime, Beijing, China). Cell apoptosis is a dynamic process, including early membrane phosphatidylserine exposure. Annexin V is a Ca^2+^ -dependent phospholipid binding protein with a high affinity for phosphatidylserine. PI was used to identify viable and non-viable cells. Apoptosis was considered to occur in cells positive for Annexin V-FITC and negative for PI. Stained cells were detected by a flow cytometer (BD Biosciences, USA). Cells were harvested after 48 h of treatment and resuspended in 1 × binding buffer (pH 7.4, 10 mM HEPES, 140 mM NaCl, 2.5 mM CaCl_2_). Then, cells were incubated with Annexin V-FITC and PI for 15 min at room temperature in the dark. Samples were operated through the flow cytometer and the results were analyzed using Cell Quest software. Cells with Annexin V−/PI+ were considered necrotic, cells with Annexin V+/PI+ were recognized as late apoptotic or secondarily necrotic, and cells with Annexin V+/PI− were counted as apoptotic. Each test was carried out at least in triplicate.

### *In Vivo* Delivery of siRNA

The C57BL/6 mice (~20 g) were injected subcutaneously with 1 × 10^6^ B16F10 cells per mouse. The volume of tumors was measured every day with a Vernier caliper after 3 days (the tumor volume was calculated approximately with the expression: Length × Width × Width/2). The tumor-bearing mice (size: 60 mm^3^) were injected subcutaneously around tumors with different formulations containing PBS only as control, cis-platinum as positive (5 mg/kg mouse; cis-platinum is a chemotherapy agent for malignant melanoma tumors) and SE-siRC (c-Myc siRNA: 0.8 mg/kg mouse; SPACE-EGF: 1.5 mg/mL) once per day or were topically daubed on tumors with SE-siRC (c-Myc siRNA: 0.8 mg/kg mouse; SPACE-EGF: 1.5 mg/mL) or c-Myc siRNA (0.8 mg/kg mouse) three times per day for 13 days. The tumor size in the treated mice was measured at the desired days after the treatment. The mice were killed and their tissues were collected after 13 days. The weight of tumors was measured and then the inhibition rate was calculated (the inhibition rate (%) = [1 - average weight of tumors in treatment group/average weight of tumors in control group] × 100%).

### Hepatotoxicity of SE-siRC

The serum was collected from the blood in the above mice after 13 days. Then, glutamic-oxalacetic transaminase (AST) and glutamic-pyruvic transaminase (ALT) were detected by a biochemical analyzer (Beckman Coulter AU480; Tokyo, Japan).

### Statistical Analysis

The data were depicted as mean ± standard deviation (S.D.) and P values were obtained using a two-tailed unpaired student’s *t* test. *P < 0.05. **P < 0.01.

## Additional Information

**How to cite this article**: Ruan, R. *et al.* Topical and Targeted Delivery of siRNAs to Melanoma Cells Using a Fusion Peptide Carrier. *Sci. Rep.* **6**, 29159; doi: 10.1038/srep29159 (2016).
